# Sarcomatoid Dedifferentiation in Renal Cell Carcinoma: From Novel Molecular Insights to New Clinical Opportunities

**DOI:** 10.3390/cancers12010099

**Published:** 2019-12-31

**Authors:** Véronique Debien, Jonathan Thouvenin, Véronique Lindner, Philippe Barthélémy, Hervé Lang, Ronan Flippot, Gabriel G. Malouf

**Affiliations:** 1 Department of Oncology, Institut de Cancérologie de Strasbourg, Hôpitaux Universitaires de Strasbourg, Université de Strasbourg, 67200 Strasbourg, France; veronique.debien@chru-strasbourg.fr (V.D.); jonathan.thouvenin@chru-strasbourg.fr (J.T.); 2 Department of Cancer and Functional Genomics, Institute of Genetics and Molecular and Cellular Biology, CNRS/INSERM/UNISTRA, 67400 Illkirch, France; p.barthelemy@icans.eu; 3 Department of Pathology, Centre Hospitalier Universitaire Régional de Strasbourg, 67200 Strasbourg, France; veronique.lindner@chru-strasbourg.fr; 4 Department of Urology, Centre Hospitalier Universitaire Régional de Strasbourg, 67000 Strasbourg, France; herve.lang@chru-strasbourg.fr; 5 Department of Cancer Medicine, Gustave Roussy, 94800 Villejuif, France; ronan.flippot@gustaveroussy.fr

**Keywords:** renal cell carcinoma, sarcomatoid, immunotherapy

## Abstract

Sarcomatoid features in renal cell carcinoma (RCC) have long been associated with dismal prognosis and poor response to therapy, while biological mechanisms underpinning sarcomatoid dedifferentiation remained obscure. Several efforts have been conducted to break down the molecular profile of sarcomatoid RCC and investigate different targeted therapeutic approaches. Mutations enriched for in sarcomatoid RCC involve, notably, *TP53*, *BAP1*, cell cycle, and chromatin-remodeling genes. The immunological landscape of these tumors is also gradually being uncovered, showing frequent expression of programmed cell death ligand-1 (PD-L1) and high levels of tumor-infiltrating lymphocytes. These features may be major determinants for the activity of immune checkpoint inhibitors in this population, which has been confirmed by retrospective studies and subgroup analyses of large randomized phase 3 trials. Combinations based on PD-1/PD-L1 inhibition have demonstrated response rates and complete responses in >50% and >10% of patients in the first-line metastatic setting, respectively, with median overall survival exceeding two years. This remarkable improvement in outcomes effectively establishes immune checkpoint inhibitor combinations as a new standard of care in patients with sarcomatoid RCC. New research fields, including epigenetic regulations and tumor–microenvironment interactions, may further sharpen understanding of sarcomatoid RCC and advance therapeutic developments.

## 1. Introduction

Renal cell carcinoma (RCC) is the third most frequent urologic malignancy, affecting more than 400,000 patients each year [1]. Approximately half of these patients will present with metastatic disease, either at diagnosis or after initial treatment of localized disease. While five-year overall survival (OS) rates do not exceed 15% in the Western world [2], vascular endothelial growth receptor (VEGFR)-directed therapies and immune checkpoint inhibitor combinations have progressively improved the prognosis of these tumors.

The landscape of RCC remains largely heterogeneous. Most RCCs (75%) consist of clear-cell subtypes, while the remaining 25% consist of different tumor subtypes grouped under the umbrella term of non-clear-cell RCC [3]. Non-clear-cell RCCs are usually more aggressive diseases than clear-cell RCCs [4] and include papillary, chromophobe, collecting duct, translocation, and medullary carcinomas. Sarcomatoid dedifferentiation is a histological feature that can be found in approximately 10% of tumors in any RCC subtype and confers aggressive behavior characterized by swift progression and dismal outcomes [5,6]; however, molecular and immunologic determinants of sarcomatoid dedifferentiation remain unclear. As survival commonly remains <12 months in patients with sarcomatoid RCC (sRCC) treated in the era of targeted molecular therapies [5], it is essential to improve our understanding of the natural history of sRCC and evaluate new therapeutic avenues in this aggressive disease.

The clinical and translational research field of sRCC is hopefully growing fast. Clinical trials of immune checkpoint inhibitors have demonstrated encouraging activity results, while an increasing number of studies have continued to provide new insights into sRCC biology. As the landscape of sRCC is being redefined, this review aims to explore the most recent advances in molecular and immune characterization of sRCC and discuss how new therapeutic developments can transform the standard of care in this population.

## 2. Pathological Implications of Sarcomatoid Renal Cell Carcinoma 

sRCC is a pathological entity defined by the presence of spindle-shaped cells in a varying proportion of the tumor area, which can account for a sarcoma-like aspect [7]. Indeed, sarcomatoid cells in RCCs have been reported to be engaged in epithelial–mesenchymal transition (EMT) [8,9], expressing mesenchymal markers including N-cadherin and vimentin, while expression of the epithelial marker e-cadherin is lost. Sarcomatoid cells also harbor increased Snail levels, a transcription factor enabling the expression of genes involved in EMT [10]. As such, the main differential diagnoses of sRCC with extensive sarcomatoid dedifferentiation are retroperitoneal leiomyosarcoma or liposarcoma. Immunohistochemistry assays may refine diagnosis, with expression of epithelial markers as keratin, cytokeratin 7, or epithelial membrane antigen (EMA) with expression of PAX8, CD10, and CAIX accounting for a renal origin [11,12]. Most often, however, sarcomatoid areas of sRCC coexist with their parent histology to make for an easier diagnosis. Rarely, some RCCs might harbor low-grade spindle cell proliferation, an entity that is distinct from sRCC and less aggressive [13]. 

Among aggressive subsets of RCCs, the International Society of Urologist Pathologists (ISUP) classification distinguishes sRCC and rhabdoid RCC^1.7^. Any of these features can be present in RCC regardless of the histological subtype, and they may coexist. The presence of sarcomatoid or rhabdoid features in any proportion would classify any clear-cell or papillary RCC as an ISUP grade 4 tumor [14]. Some sRCCs with extensive sarcomatoid dedifferentiation encompassing the whole tumor without clear evidence of any parent histology may be considered as unclassified RCCs [15]. The occurrence of sarcomatoid dedifferentiation may vary according to the parent histology. Sarcomatoid features have thus been reported in 9% of chromophobe RCCs, 5% of clear-cell RCCs, and 2% of papillary RCCs in a large retrospective study [16]. 

The aggressiveness of sRCC is highlighted by the high frequency of distant metastases at diagnosis, reported in approximately two-thirds of patients with sRCC of any histology compared to approximately 30% of patients without sarcomatoid features [17,18]. The proportion of sarcomatoid features also plays a role in the definition of prognosis, as a higher percentage has been shown to be associated with higher risk of relapse in a localized setting, and worse overall survival [17,19]. While the quantification of sarcomatoid features is an important prognostic indicator, accurate evaluation might be compromised by the extent of tumor heterogeneity. It has been shown that biopsies are up to two-fold less likely to identify sarcomatoid features compared to the analysis of nephrectomy specimens [20], while some tumors may harbor sarcomatoid features exclusively in distant metastases [19], which may lead to an underestimation of the prevalence of sRCC in kidney cancer patients. It is yet unknown whether sarcomatoid dedifferentiation can also occur as a late event upon disease progression, an element that could have prognostic and therapeutic implications for late-stage disease.

## 3. Molecular Landscape of Sarcomatoid Renal Cell Carcinoma

Multiple studies have aimed to unravel the molecular picture of sRCC and events leading to sarcomatoid dedifferentiation, an important effort considering the low incidence of these tumors and the heterogeneity of parent histologies. The theory of a common cell of origin for the epithelial and sarcomatoid components in sRCC has been confirmed by several studies. As demonstrated in sRCC of clear-cell type, sarcomatoid and epithelial components share most copy number alterations, X chromosome inactivation patterns, and single nucleotide variants [21,22,23]. Notably, clear-cell sRCC harbor a lower frequency of 3p loss, locus of the *VHL* gene, but also of chromatin remodeling genes *BAP1* and *PBRM1* [22,24]. More surprisingly, clear-cell sRCC are also devoid of 9p and 14q alterations usually associated with poor prognosis and high grade in clear-cell RCC [22]. 

Likewise, the transcriptomic profile of clear-cell sRCC harbors differences compared to that of nonsarcomatoid clear-cell RCC, with activation of pathways involved in aggressiveness and epithelial mesenchymal transition. It has been shown that clear-cell sRCCs harbor higher expression of VEGF and TGFβ1 pathways, while the TP53 pathway is repressed [22,24]. Clear-cell sRCC expression profile is also enriched in genes involved in the poor prognostic signature ccB [25] compared to non sarcomatoid clear-cell RCC, consistent with their clinical aggressiveness [22]. A few studies have pinpointed differences between the transcriptional profiles of sarcomatoid and epithelial components in a single tumor [22,23,24]. These have shown that several genes involved in EMT may have increased expression in the sarcomatoid component of clear-cell sRCC, which could account for the mesenchymal phenotype of these cells [22]. Additional insights from an independent cohort showed that sarcomatoid components might harbor increased Aurora kinase-1 expression, suggested to drive malignancy by increasing mammalian target of rapamycin (mTOR) activation [26].

**Figure 1:**
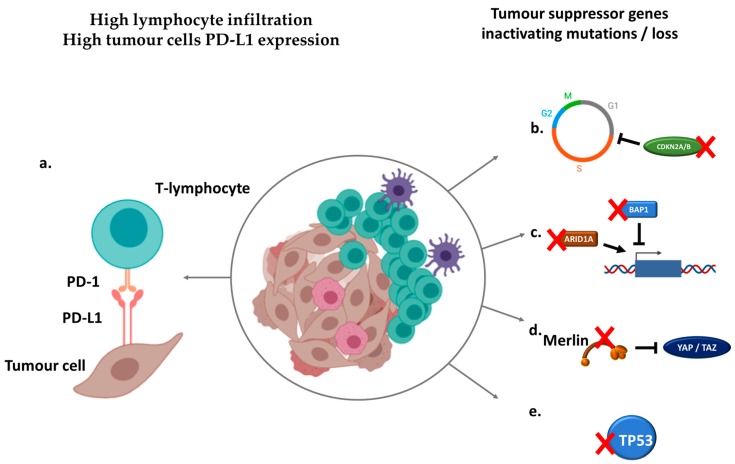
Immunologic and genomic hallmarks of sarcomatoid dedifferentiation in renal cell carcinoma (RCC). (**a**) Sarcomatoid renal cell carcinomas (sRCCs) are associated with higher programmed cell death ligand-1 (PD-L1) expression on tumor cells and higher lymphocyte infiltration. (**b**) Recurrent alterations of cell cycle inhibitors *CDKN2A/B* promote cell proliferation and epithelial/mesenchymal transition. (**c**) Loss of chromatin-remodeling genes *BAP1* and *ARID1A* induce genome-wide expression deregulation. (**d**) Loss of Merlin, encoded by the *NF2* gene, promotes Hippo pathway activation, leading to growth and aggressiveness. (**e**) Loss of tumor suppressor gene *TP53* favors survival and proliferation.

More differences may be found in exploring the genomic alterations of sRCC, which reveals several potential drivers of sarcomatoid dedifferentiation (Figure 1). A study of 26 sRCCs using tumor microdissection from mixed parent histologies by targeted sequencing showed that sRCC harbored frequent mutations in *TP53*, *VHL*, *CDKN2A*, and *NF2* in 42%, 35%, 27%, and 19% of tumors, respectively [27]. *TP53* mutations were not associated with a specific histological subtype and were significantly enriched compared to non sarcomatoid RCC cohorts as those were found in only 2% of clear-cell RCC from the Cancer Genome Atlas (TCGA) dataset [28]. Likewise, *NF2* mutations only involved 1% of clear-cell RCC from the TCGA. Additional studies have depicted the mutational landscape of sRCC with focus on specific histologies. Whole-exome sequencing of sRCC from clear-cell origin confirmed the high prevalence of *TP53* alterations in two independent cohorts [23,24]. Additional recurrent mutations in sRCC from clear-cell origin include Hippo regulators *FAT1/2/3* and chromatin remodeling gene *ARID1A* [23] as well as tumor suppressor *PTEN* and TGFβ regulator *RELN* [24]. Comparison of sarcomatoid and epithelial components of clear-cell sRCC hint at a higher mutational burden in the sarcomatoid component and a higher frequency of *TP53*, *BAP1*, and *ARID1A* mutations [23]. Mutations in those three genes have been described as mutually exclusive, suggesting potential driver events [23]. *TP53* alterations have also been described in sRCC from papillary origin, along with alterations of Hippo member *NF2*, while mutations in *RELN* are reported to be enriched in sRCC regardless of the parent histology [24].

While these studies do not provide a unique explanation for the emergence of sarcomatoid features, recurrent mutations might participate in driving this aggressive phenotype, along with other deregulations of cellular processes. Likewise, an updated analysis of the TCGA dataset identified a subset of metabolically divergent chromophobe RCC, characterized by low expression of genes involved in the Krebs cycle, the electron transport chain, repression of the AMPK, and overexpression of genes involved in the ribose synthesis [29]. This signature was associated with poor outcomes and, strikingly, four of the six patients (67%) with metabolically deficient chromophobe RCC had a disease that presented with sarcomatoid dedifferentiation. Other particular phenotypes may include hypermutated tumors, which was found in 2 of 21 (10%) clear-cell sRCC in a single institution cohort [23]; this phenotype had not been encountered in the larger, non-sRCC TCGA dataset. This hypermutated phenotype was due to somatic *MSH2* and *POLE* mutations, which could have favored the emergence of the sarcomatoid phenotype in these tumors.

A better understanding of sarcomatoid transformation may also be achieved by studying aggressive unclassified RCC (uRCC), which may include tumors with an exclusive sarcomatoid or rhabdoid component [15]. A molecular study of 62 uRCC identified a *NF2*-deficient subgroup encompassing 26% of tumors and characterized by worse outcomes [30]. This subgroup of tumors also displayed more frequent *SETD2* alterations and 3p loss. As such, alterations of the Hippo pathway may be an important event for tumor aggressiveness and progression regardless of pathological features of RCC, which may have translational and therapeutic relevance for targeted approaches [31].

Several aspects of sRCC as a disease remain unknown. The relationship between molecular heterogeneity and response to therapy is yet to be defined, while the natural history of the disease may also be heavily influenced by the tumor microenvironment. In the era of immune checkpoint inhibitors, immune infiltration and exploration of immune markers will be key factors for the management of sRCC.

## 4. The Immune Microenvironment of Sarcomatoid Renal Cell Carcinoma

**Figure 2:**
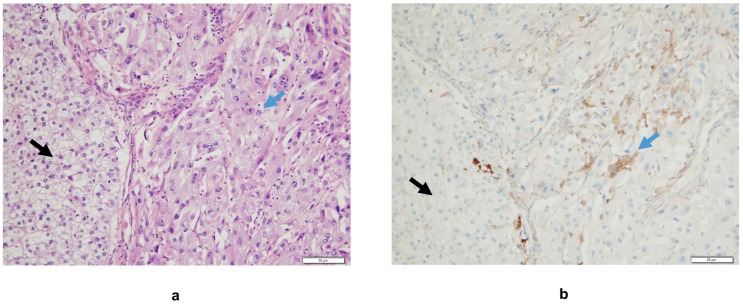
(**a**) Interface between areas of classical clear-cell carcinoma (black arrow) and its sarcomatoid component (blue arrow), Hematoxylin Eosin staining. (**b**) A higher density of tumor-infiltrating mononuclear inflammatory cells expressing PD-L1 is observed in the sRCC area compared to the clear-cell RCC one (monoclonal mouse antibody clone 22C3, hematoxylin counter staining). In this case, there is no PD-L1 expression on tumor cells.

The biology of sRCC may account for a particular immune context when compared to non-sRCC (Figure 1). The expression of the immune checkpoint programmed cell death ligand-1 (PD-L1), promoting immune tolerance and targeted by several immune checkpoint inhibitors, is increased on the surface of sRCC cells compared to non-sRCC ones (Figure 2) regardless of parent histology and non-sRCC tumor grade [32,33]. Interestingly, there is a clear difference in PD-L1 expression between the different components of sRCC. While sarcomatoid components of sRCC display the highest PD-L1 expression, levels of PD-L1 expression on epithelial components of sRCC are similar to those of non-sRCC [32]. As such, PD-L1 expression levels are associated with the extent of sarcomatoid dedifferentiation [33]. Data from prospective clinical trials have confirmed the high levels of PD-L1 expression on sRCC of clear-cell type, with ≥50% of patients displaying PD-L1 expression ≥1% on tumor cells [34] or immune-infiltrating cells [35,36]. 

sRCC also present with a higher density of tumor-infiltrating lymphocytes (TILs) compared to non-sRCC regardless of histology (Figure 2), and most of these lymphocytes have been reported to express PD-1 [33]. Up to 40% of sarcomatoid components of 118 sRCC from any histology were reported to harbor both PD-L1 expression and TIL infiltration in a single center study, compared to 8% of epithelial components of sRCC and 1% of control clear-cell RCCs [32]. This pattern has been suggested to be associated with immune resistance and may confer sensitivity to immune checkpoint inhibitors. This is corroborated by the high concomitant expression of PD-L1 in tumor cells and PD-1 in TIL, which was reported in up to 50% of sRCC of any subtype compared to less than 5% of non-sRCC in another retrospective cohort, suggesting that the PD-1/PD-L1 axis is active in sRCC [33]. 

Molecular studies provide additional basis for the immunogenic potential of sRCC. Study of gene expression signatures in the IMmotion151 phase 3 trial of atezolizumab plus bevacizumab versus sunitinib reported that clear-cell sRCC had a higher T-effector signature (54% versus 40%), a lower angiogenesis signature (34% versus 65%), and a higher expression of PD-L1 (63% versus 39%) compared to nonsarcomatoid clear-cell RCC [37]. Along with concordant clinical outcomes showing potent activity of atezolizumab plus bevacizumab in this population (Table 1), these data corroborate the immunogenicity of these tumors and the potential for immune-directed approaches. 

Tumor mutational burden is another hallmark that has been suggested to predict outcomes to immunotherapy in solid tumors through the formation of immunogenic neoantigens that would elicit antitumor immune response [38]. Data on sRCC remain equivocal. In a large retrospective study based on targeted sequencing of 79 sarcomatoid or rhabdoid RCC from any parent histology, mutational burden was not significantly different from nonsarcomatoid and nonrhabdoid tumors [39]. While a higher rate of somatic mutations has been described in unselected sRCC from another small dataset, these did not significantly affect relevant cancer-related genes [24]. Some data suggest that *TP53* alterations may be associated with higher mutational load, but the clinical relevance is yet unclear [27]. 

Overall, there is consistent data demonstrating that sRCC may constitute a more immunogenic subtype of cancer than non-sRCC regardless of histology, which provide an interesting basis for the study of immune checkpoint inhibitors in these populations. Additional exploration of the immune microenvironment [40], specific mutation profiles including small insertions and deletions [41], and expression of other immune checkpoints [42] should be pursued to improve our understanding of sRCC immunity. 

## 5. Improving Therapeutic Strategies through Immune Checkpoint Inhibition

Therapeutic management of sRCC shares similarities with that of non-sRCC. In a context of nonresectable or metastatic disease, patients have so far been treated with systemic therapies based on VEGFR-targeted agents. Data from patients treated with sunitinib and sorafenib, however, show very limited efficacy, with objective response rates (ORR) below 20% and median progression-free survival (PFS) and OS below 6 and 12 months, respectively [43]. Additional chemotherapy-based strategies have been used, assuming that antimitotic agents would be effective against this highly proliferative disease. In phase 2 trials, combination of gemcitabine with doxorubicin [44] as well as gemcitabine plus capecitabine and bevacizumab [45] demonstrated some activity regardless of the histological subtype of sRCC, with ORR between 16% and 20%, PFS of 3.5 to 5.5 months, and OS of 8.8 to 12 months. More recently, the combination of gemcitabine and sunitinib showed mild antitumor activity in unselected sRCC with an ORR of 26% and PFS and OS of 5 and 10 months, respectively. Subgroup analyses showed an improved response rate in tumors with more than 10% of sarcomatoid features and improved survival in patients with poor-risk disease [46]. 

These poor outcomes may be improved by immune checkpoint inhibitors, which has become the standard of care in patients with clear-cell RCC in monotherapy or combination. Notably, nivolumab plus ipilimumab [47] and pembrolizumab plus axitinib [48] have both demonstrated improved OS in intermediate/poor risk patients and all comers, respectively, while atezolizumab plus bevacizumab [37] and avelumab plus axitinib [47] demonstrated PFS improvement compared to sunitinib in the first-line metastatic setting. First subgroup analyses, including patients with sarcomatoid tumors, are being reported with promising efficacy results (Table 1). 

The Keynote-426 trial assessing the pembrolizumab plus axitinib combination and the JAVELIN Renal 101 trial of avelumab plus axitinib both demonstrated improved outcomes in patients with clear-cell sRCC compared with sunitinib [36,49]. The ORR with those combinations were strikingly high at 59% and 47%, with complete responses (CR) in 12% and 4% of patients, respectively. These data translated into increased PFS compared to sunitinib with hazard ratios (HR) of 0.54 (95% confidence interval (CI) 0.29–1.00) and 0.57 (95% CI 0.32–1.00) for OS, respectively. Similarly, interesting results were reported with the IMmotion151 trial, which evaluated the combination of atezolizumab plus bevacizumab in the same population. An ORR of 49%, including a complete response rate of 10%, and a HR for PFS of 0.52 (95% CI 0.34–0.79) and 0.64 (95% CI 0.41–1.01) in sRCC were reported [35]. 

The combination of two immune checkpoint inhibitors may also provide impressive results in sRCC patients, as demonstrated by the CheckMate-214 study of nivolumab plus ipilimumab. In patients with intermediate or poor-risk clear-cell sRCC, PFS and OS were improved in the experimental arm, with HR of 0.61 (95% CI 0.38–0.97) and 0.55 (95% CI 0.33–0.90), respectively, compared to sunitinib. Importantly, the ORR achieved by the combination of nivolumab plus ipilimumab was as high as 57%, with an impressive proportion (18%) of patients achieving complete response (Table 1). These results are encouraging as complete response to immunotherapy has been recently suggested to be a potential surrogate marker for very long-term survival [50].

Additional data stems from phase 2 trials spanning across uncommon histologies. In a phase 2 trial of atezolizumab plus bevacizumab, patients with clear-cell RCC and sarcomatoid dedifferentiation >20% had an ORR of 50% [34]. Data from the cohort B of the Keynote-427 study evaluating pembrolizumab monotherapy showed an ORR of 42% in patients with sRCC of non-clear-cell subtypes [51].

Prospective clinical trial data thus show convincing efficacy of immune checkpoint inhibitors in sRCC patients and is in line with translational work suggesting that sRCC may be immune-reactive tumors. The use of immunotherapy combinations should be considered as standard of care in these populations, while biomarker analyses are awaited to better inform patient selection. 

## 6. Perspectives

The study of sRCC has become a dynamic field, sparking hope for sustained improvement in outcomes in this aggressive subset of tumors. It is now established that sRCC are immunologically “hot” tumors that demonstrate excellent responses to immune checkpoint inhibitors and prolonged survival with combination-based strategies. Current efforts to unravel the molecular landscape of these tumors might help develop targeted strategies to overcome resistance to current therapies.

Many questions about sRCC biology have yet to be answered to get to this point. The mechanisms underlying sarcomatoid dedifferentiation are still mostly unknown. Despite evidence of recurrent genomic alterations, those are not specific to sRCC and may not be enough to drive this aggressive phenotype. In addition, the relevance of these alterations for targeted therapies has yet to be investigated in dedicated clinical trials. More answers could lie in epigenetics and regulatory processes that trigger transcriptional programs involved in sarcomatoid transformation. Such processes may be impacted by the cellular context, including alterations of cellular metabolism [29] as well as interactions between tumor cells and their surrounding microenvironment [52]. The noncoding genome also needs to be explored in the future. In particular, alterations affecting regulatory RNAs may impact cell machinery and disrupt gene expression toward epithelial–mesenchymal transition [53]. The impact of parent histologies on such cellular processes also needs to be determined as the diversity of RCC subtypes may account for wide genetic and epigenetic variations.

The encouraging results of immune checkpoint inhibitor combinations in a disease known to be refractory to past standard of care therapies brings new optimism in the field. Biomarkers of response to therapy remain to be found, similar to non-sRCC, where PD-L1 expression [54], tumor mutational burden [38], or gene expression profiles [55] have yet to prove their clinical utility to accurately stratify patients. Additional combinations using modified proinflammatory cytokines [56] or novel checkpoint inhibitors [57] have the potential to further improve the efficacy of PD-1/PD-L1-based regimens. Approaches based on multimodal therapy, such as combining stereotactic radiation therapy to bolster immunity, could be interesting to explore in these inflamed tumors [58]. Opportunities for targeted therapies should also be evaluated: cell cycle inhibitors could be interesting in tumors with alterations of cell cycle proteins [59]; approaches targeting chromatin remodeling complexes in tumors already deficient in one or multiple chromatin-remodeling genes, such as *ARID1A*, could promote synthetic lethality, as demonstrated in other solid tumors [60]; *NF2*-deficient tumors could be targeted in preclinical models by YAP/TAZ depletion associated with MEK inhibition [31]. The progress made in the metastatic setting may also translate into localized disease. Multiple trials are ongoing for evaluating immune checkpoint inhibition perioperatively, including nivolumab (NCT03055013) and pembrolizumab (NCT03142334), which could be beneficial to these tumors at high risk of recurrence. 

## 7. Conclusions

Clinical and translational research efforts have transformed the understanding of sRCC: from a hard-to-treat disease with limited biological understanding, it is becoming part of a burgeoning research field with major advances in outcomes and new paths to innovative therapeutic developments. Evaluation of novel treatment strategies and potential biomarkers of response to therapy are needed to improve patient selection and the prognosis of this aggressive disease.

## Figures and Tables

**Table 1:** Activity of immune checkpoint inhibitors in clear-cell sRCC from subgroup analyses of phase 3 trials.

Trials	Population	Agents	N	ORR	CRR	Median PFS	Median OS
**Keynote-426**	Intent-to-treat	pembrolizumab + axitinib	51	59%	13%	8.4 months	NR
		vs.	vs.	vs.	vs.	vs.	vs.
		sunitinib	54	32%	2%	NR	NR
**CheckMate-214**	IMDC poor or intermediate risk	nivolumab + ipilimumab	60	57%	18%	8.4 months	31.2 months
		vs.	vs.	vs.	vs.	vs.	vs.
		sunitinib	52	19%	0%	4.9 months	13.6 months
**IMmotion151**	Intent-to-treat	atezolizumab + bevacizumab	68	49%	10%	8.3 months	21.7 months
		vs.	vs.	vs.	vs.	vs.	vs.
		sunitinib	74	14%	3%	5.3 months	15.4 months
**JAVELIN Renal 101**	Intent-to-treat	avelumab + axitinib	47	47%	4%	7.0 months	NA
		vs.	vs.	vs.	vs.	vs.	
		sunitinib	61	21%	0%	4.0 months	

Abbreviations: ORR: objective response rate, CRR: complete response rate, PFS: progression-free survival, OS: overall survival, NR: not reached, NA: not available, CPS: combined positive score.
